# Estimation of the Undiagnosed Intervals of HIV-Infected Individuals by a Modified Back-Calculation Method for Reconstructing the Epidemic Curves

**DOI:** 10.1371/journal.pone.0159021

**Published:** 2016-07-12

**Authors:** Ngai Sze Wong, Ka Hing Wong, Man Po Lee, Owen T. Y. Tsang, Denise P. C. Chan, Shui Shan Lee

**Affiliations:** 1 Stanley Ho Centre for Emerging Infectious Diseases, The Chinese University of Hong Kong, Hong Kong, China; 2 Institute for Global Health & Infectious Diseases, University of North Carolina at Chapel Hill, Chapel Hill, NC, United States of America; 3 University of North Carolina Project-China, Guangzhou, Guangdong, China; 4 Special Preventive Programme, Department of Health, Hong Kong Special Administrative Region Government, Hong Kong, China; 5 Department of Medicine, Queen Elizabeth Hospital, Hong Kong, China; 6 Department of Medicine and Geriatrics, Princess Margaret Hospital, Hong Kong, China; University of Malaya, MALAYSIA

## Abstract

**Background:**

Undiagnosed infections accounted for the hidden proportion of HIV cases that have escaped from public health surveillance. To assess the population risk of HIV transmission, we estimated the undiagnosed interval of each known infection for constructing the HIV incidence curves.

**Methods:**

We used modified back-calculation methods to estimate the seroconversion year for each diagnosed patient attending any one of the 3 HIV specialist clinics in Hong Kong. Three approaches were used, depending on the adequacy of CD4 data: (A) estimating one’s pre-treatment CD4 depletion rate in multilevel model;(B) projecting one’s seroconversion year by referencing seroconverters’ CD4 depletion rate; or (C) projecting from the distribution of estimated undiagnosed intervals in (B). Factors associated with long undiagnosed interval (>2 years) were examined in univariate analyses. Epidemic curves constructed from estimated seroconversion data were evaluated by modes of transmission.

**Results:**

Between 1991 and 2010, a total of 3695 adult HIV patients were diagnosed. The undiagnosed intervals were derived from method (A) (28%), (B) (61%) and (C) (11%) respectively. The intervals ranged from 0 to 10 years, and were shortened from 2001. Heterosexual infection, female, Chinese and age >64 at diagnosis were associated with long undiagnosed interval. Overall, the peaks of the new incidence curves were reached 4–6 years ahead of reported diagnoses, while their contours varied by mode of transmission. Characteristically, the epidemic growth of heterosexual male and female declined after 1998 with slight rebound in 2004–2006, but that of MSM continued to rise after 1998.

**Conclusions:**

By determining the time of seroconversion, HIV epidemic curves could be reconstructed from clinical data to better illustrate the trends of new infections. With the increasing coverage of antiretroviral therapy, the undiagnosed interval can add to the measures for assessing HIV transmission risk in the population.

## Introduction

Before progression to AIDS, HIV infection is largely asymptomatic in the period since seroconversion, the duration of which can be as long as 7 years or more in the absence of treatment.[1] An HIV-infected individual remains undiagnosed, unless he/she receives an HIV test for different reasons. Within this undiagnosed period, infected individuals are not aware of their HIV status. Their transmission risk can be substantial in the presence of a high partner exchange rate and the practice of unprotected sex. After HIV diagnosis, transmission risk may fall as a result of self-initiated reduction of risk behaviours and/or interventions.[2] Moreover, good coverage of highly active antiretroviral treatment (HAART) could reduce the population viral burden, thereby minimizing the transmission risk, as concluded in the HPTN052 study.[3, 4] Therefore, the status of being undiagnosed, the first stage of the care continuum cascade, constitutes a major gap for achieving effective interventions through HAART. Epidemiologically, the lag time between infection and diagnosis is an obstacle for proper interpretation of epidemic curves plotted by annual numbers of new HIV diagnoses, as recent and past infections could not be differentiated. Quantification of the undiagnosed intervals is, therefore instrumental for reconstructing epidemic curves for supporting the effective monitoring of the epidemic and evaluating interventions introduced.

In the past, HIV incidence back-calculated by computing the number of diagnosed AIDS cases and distribution of incubation period between HIV infection and AIDS diagnosis[5] was a reasonable approach. The widespread use of HAART since the mid-1990s has however distorted the natural history of HIV/AIDS. While a few studies have introduced modified back-calculation method that incorporated diagnosed HIV cases,[6] the estimation of new infections was often made at aggregate level. Other studies have used biological approaches such as tests for recent infection (TRIs), recent infection testing algorithm (RITA) and BED HIV-1 Capture Enzyme Immunoassay to determine whether a diagnosed individual was recently infected.[7, 8] However, such method was limited by the availability of samples, technologies and resources, and could only broadly distinguish between recent and non-recent infections. To date, some studies have estimated the prevalence of undiagnosed HIV-infected individuals and investigated their epidemiological characteristics, as reported in China,[9] India,[10] Spain, Italy, Slovakia, Romania, Slovenia and Czech Republic.[11] While these studies have provided insights into the size of the hidden infective populations, temporal patterns were not systematically evaluated.

At an individual level, the undiagnosed interval has largely been ignored so far, except for a limited number of studies which computed seroconverters’ pre-treatment CD4 depletion rate to back-calculate the year of seroconversion,[12, 13] or used diagnosed individuals’ specific test-seeking behaviours and clinical status at diagnosis to estimate the time interval between infection and diagnosis.[14, 15] Against these backgrounds, we propose to expand the modified back-calculation method to make full use of currently available clinical data to determine the undiagnosed interval for each HIV positive individual, using seroconverters’ CD4 depletion as the reference group With this approach, we estimated the seroconversion year and undiagnosed interval at individual level and reconstructed the HIV incidence curves to help explain the temporal trend of virus transmission in the city of Hong Kong.

## Methods

### Data source

In Hong Kong, 3 HIV specialist clinics in the public service are providing care to almost all reported HIV/AIDS cases in the territory, the data from which constituted the cohort database described in this study.[16] HAART is initiated in accordance with local guidelines adapted from the recommendations of Department of Health and Human Services in USA.[17] Demographics, baseline clinical conditions and regularly collected laboratory measures (CD4 and viral load) in 1985–2012 were accessed. Approvals from the Research Ethics Committee of the Joint Chinese University of Hong Kong—New Territories East Cluster, and Kowloon Central/Kowloon East Cluster of the Hospital Authority were obtained. In compliance with the Personal Data (Privacy) Ordinance, data access was approved by the Department of Health, Hong Kong Special Administrative Region Government. No informed consent was obtained as the collected data were anonymized and collected retrospectively. This study was conducted in compliance with Declaration of Helsinki.

### Modified back-calculation methods for estimating seroconversion year

In our study, the annual number of new infections, total number of undiagnosed infections, and every HIV infected person’s undiagnosed interval were derived after estimating the seroconversion year individually. Estimation was made by the modified back-calculation approach, taking reference from the pre-treatment CD4 trajectory of known HIV-infected patients. As a set-point viral load is reached about 6 months after infection,[1] this interval was deducted from the time-point when CD4 count reaches a plateau following acute infection. According to the number of pre-treatment CD4 measurements in the clinical dataset, patients were classified into Group A (>3 CD4 counts), Group B (1–3 CD4 counts) and Group C (no CD4 count) for the estimation.

#### Group A (individuals with >3 pre-treatment CD4 counts)

Methodologically, if over 3 pre-treatment CD4 counts have been recorded, the levels were input in multilevel model (lme4 R package) to derive the pre-treatment CD4 depletion rate for each patient. Linear multilevel model was performed because of the repeated CD4 measurements available per patient.[18] In the model, CD4 measurements (outcome variable) were nested by patients, with time (defined as months from the first CD4 measurement after diagnosis) as random intercept and random slope, and ethnicity and gender as random intercepts. The seroconversion year of HIV patients who were infected via injection drug use or sexual transmission were estimated separately in multilevel model. Different combination of variables in multilevel model were explored and the best multilevel model was selected with reference to intraclass correlation coefficient (ICC).[19] By randomly selecting a CD4 count within the normal reference range of a healthy adult for 1000 times,[20–22] and using these as the starting points for CD4 depletion, the seroconversion year of a patient was calculated in Eq 1 [23, 24] for 1000 simulations:

$$seroconversion\;year=\;diagnosis\;year+\;\frac{random\;normal\;CD4\;reference\;range_{i,j}-a}{monthly\;CD4\;slope*12}$$ (1)

Where **i** is gender (male, female), **j** is ethnicity (1. Asian, 2. White, 3. African & others),[20–22] **a** is intercept of an individual’s regression line in multilevel model, **CD4 slope** is the adjusted coefficient of the individual’s regression line in multilevel model

The simulation results falling outside the lower boundary (a. the last negative HIV testing year; or b. 1980, which might be the year with the first possible infection case in Hong Kong; or c. year of attaining 12 year-old, possible minimum age of being sexually active) and the upper boundary (year of HIV diagnosis) were discarded.

We have selected the pre-treatment CD4 counts of seroconverters (patients with ≤2 years’ time interval from last negative HIV test to first positive HIV test) to form a reference dataset to validate the simulation results. Their CD4 measurements were input to the same multilevel model as patients in Group A, with the removal of the year of last negative HIV testing date as the lower boundary of filter. The simulation results were compared with the mid-point of the interval between the last negative and the first positive HIV tests, assuming the latter method gave the most precise estimation.[25] The seroconversion time of patients in Group A was derived from the third quartile of the simulation results as these were closest to the mid-point estimation by comparison. (S1 Table) The discrepancy would be the smallest if we take the 90th percentile, but this would not be meaningful unless everyone had seroconverted a year before diagnosis.

#### Group B (individuals with 1–3 pre-treatment CD4)

Multilevel models were performed in a different way for patients with 1–3 CD4 measurements before treatment initiation, or if Group A estimation failed due to minimal changes in CD4 count pre-treatment or rising pre-treatment CD4 count with slope of depletion above -1/μL/month. Reference range of coefficients showing the relationship between seroconversion time (mid-point of interval between last negative and first positive HIV testing ≤2 years) and CD4 count was first established from seroconverters in the same cohort. The seroconversion year of injection drug users (IDU) and sexually acquired HIV patients were estimated separately by linear multilevel model. Time (months from seroconversion time) and ethnicity (being White or not) were random intercepts in the model. Gender was not included in the selected multilevel model as it was not significantly associated with CD4 count. With the coefficients determined in multilevel model, the year of seroconversion was estimated by:

$$\text{CD}4\text{ measurement year }-\;\frac{\text{CD}4\;-\text{ random intercept }-\text{ coefficient*White}}{12\text{*random CD}4\text{ slope}}$$ (2)

The randomly generated intercept and coefficient were derived from the mean and standard deviation of coefficients calculated in multilevel model. A total of 2500 simulations were performed for a clinical measurement available. As the seroconversion time of patients in Group A were estimated by the third quartile of simulation result, the same logic applied here.

#### Group C (individuals without pre-treatment CD4 count)

For individuals without pre-treatment CD4 count or with flat or rising pre-treatment CD4 slope (above -1/μL/month), estimation of seroconversion year was made from the estimation results of Group B. This was based on the distribution of estimated undiagnosed interval (from year of seroconversion to diagnosis) in patients in Group B, as the in care condition between the two Groups were similar, both with less frequent or irregular visit to the clinics before treatment. Due to their similarity, the seroconversion year was calculated by subtracting the median undiagnosed interval from the year of diagnosis, stratified by mode of transmission and status of late HIV diagnosis.(Table 1) Late HIV diagnosis here refers to the diagnosis of AIDS within 3 months of HIV diagnosis. Sensitivity analyses were performed to examine the difference of seroconversion estimation results between selection of reference groups 1) Group A, 2) Group B and 3) Group A and B that were used to derive undiagnosed intervals, and selection of central tendency (a. median, b. first quartile and c. third quartile) of undiagnosed intervals.

**Table 1:** Summary for median of intervals between HIV diagnosis year and 3^rd^ quartile of the simulation results of seroconversion year in Group B patients.

Subgroups by transmission route	Median of interval (year)	n	Median of interval (year)	n
	Non-late diagnosis		Late diagnosis*	
Blood recipient	1	39	1	8
Heterosexual female	2	333	3	166
Heterosexual male	2	518	3	499
Injection drug use	2	148	2	35
Men who have sex with men	2	739	3	295
Undetermined	3	37	4	31

*Late diagnosis—patients diagnosed with AIDS within 3 months of HIV diagnosis

#### Analyses

With the estimation of seroconversion year at individual level, we plotted the HIV incidence curves as the yearly estimated count of individuals who had seroconverted. The annual number of undiagnosed individuals were plotted as the total number of HIV-infected remaining undiagnosed in the respective year. Undiagnosed interval was defined as the difference between year of seroconversion and year of diagnosis. The temporal trend of diagnosed, newly diagnosed, newly infected and undiagnosed were smoothed in 2-year windows by Seasonal-Trend Decomposition Procedure based on Loess in R3.2.2.[26] Associations between characteristics of patients and undiagnosed interval >2 years were examined in univariate analyses. We examined the confounding effects of ethnicity (Chinese vs non-Chinese), gender (female vs male), age at diagnosis (continuous variable) and mode of transmission (MSM vs non-MSM) in multivariable logistic regression models in SPSS. Confounding variables affecting >10% change of crude odds ratio (OR) of other independent variables were kept in the model for calculating adjusted odds ratio (aOR) of other independent variables. Patients were selected for analysis if they were diagnosed in 1991–2010, during which a CD4-guided approach was the norm for treatment initiation. We also used an alternative approach to estimate seroconversion time in sensitivity analysis. First, we estimated the seroconversion time with the original method proposed in this study. Second, we used Group B method to estimate the seroconversion time for patients with any pre-treatment CD4 counts (i.e. patients in Group A and Group B), while Group C method for Group C patients. Third, we used Group C method to estimate the seroconversion time for all patients. Lastly, we took the average undiagnosed interval among the 3 estimations for each patient, and performed univariate analysis to examine the association between long undiagnosed interval and independent variables.

## Results

As of 2012, 74,541 clinical measurements of 4551 HIV patients have been collected, of which data from 3695 adult (aged 18 or older) HIV patients diagnosed in 1991–2010 were selected for inclusion in the study. The sample size accounted for about 80% of diagnosed HIV cases recorded in the surveillance system during the 20-year period. Among them, 27% (999/3695) were in late HIV diagnosis, and 14% (528/3695) were seroconverters with an interval ≤2 years between last HIV negative testing date and first HIV positive testing date. The seroconversion year of each case was estimated, of which 1050 (28%) were in Group A, 2241 (61%) in Group B and 404 (11%) in Group C.

### Multilevel model results

Linear multilevel models were performed to estimate pre-treatment CD4 depletion rate in both Group A and Group B. There was high heterogeneity of CD4 depletion rate among patients, with ICC ranging between 51% and 89% (Table 2). In Group A, the CD4 depletion rate of patients who contracted HIV through sexual contact (n = 831 patients) and contaminated needle sharing (n = 95 patients) were similar at around –5 cells/μL per month from baseline. Among individuals infected through sexual contact, the White were adjusted to have higher CD4 (82.7/μL) than their counterparts. In Group B, pre-treatment CD4 depletion rate among seroconverters infected through sexual contact (n = 347 patients) was similar to those in Group A. However, the CD4 depletion rate of IDU seroconverters (n = 8 patients) was lower (- 2/μL) than those in Group A. The third quartile of simulation results in Group A and Group B are shown in S1 Fig.

**Table 2:** Pre-treatment CD4 depletion rate in cells/month estimated in the linear multilevel models for patients in Group A and Group B.

	sexually acquired infections	infections in IDU
**Group A**		
*Dataset for multilevel model*: *CD4 data of patients with >3 pre-treatment CD4 count*		
**Sample size**	8398 CD4 counts in 831 patients	728 CD4 counts in 95 patients
**ICC**	86%	89%
**Random effect**		
Months from baseline CD4	variance = 13.4, SD = 3.7	variance = 4.5, SD = 2.1
**Fixed effect**		
Months from baseline CD4	coe: -5.0 (s.e. = 0.16, t-value = -0.7)	coe: -4.2 (s.e. = 0.4. t-value = -11.9)
Being White	coe: 82.7 (s.e. = 28.6, t-value = 2.9)	/
**Group B**		
*Dataset for multilevel model*: *CD4 data of seroconverters*		
**Sample size**	2971 CD4 counts in 347 patients	72 CD4 counts in 8 patients
**ICC**	84%	51%
**Random effect**		
Months from seroconversion	variance = 17.8, SD = 4.2	variance = 0.3, SD = 0.5
**Fixed effect**		
Months from seroconversion	coe: -4.7 (s.e. = 0.3. t-value = -15.2)	coe: -2.2 (s.e. = 0.5. t-value = -4.6)
Being White	coe: 73.5 (s.e. = 30.1, t-value = 2.4)	/

ICC (intraclass correlation coefficient) = intercept variance / (intercept variance + residual variance) [19] coe-coefficient, s.e.-standard error

### Trends of annual number of new infections and undiagnosed infections

**Fig 1:**
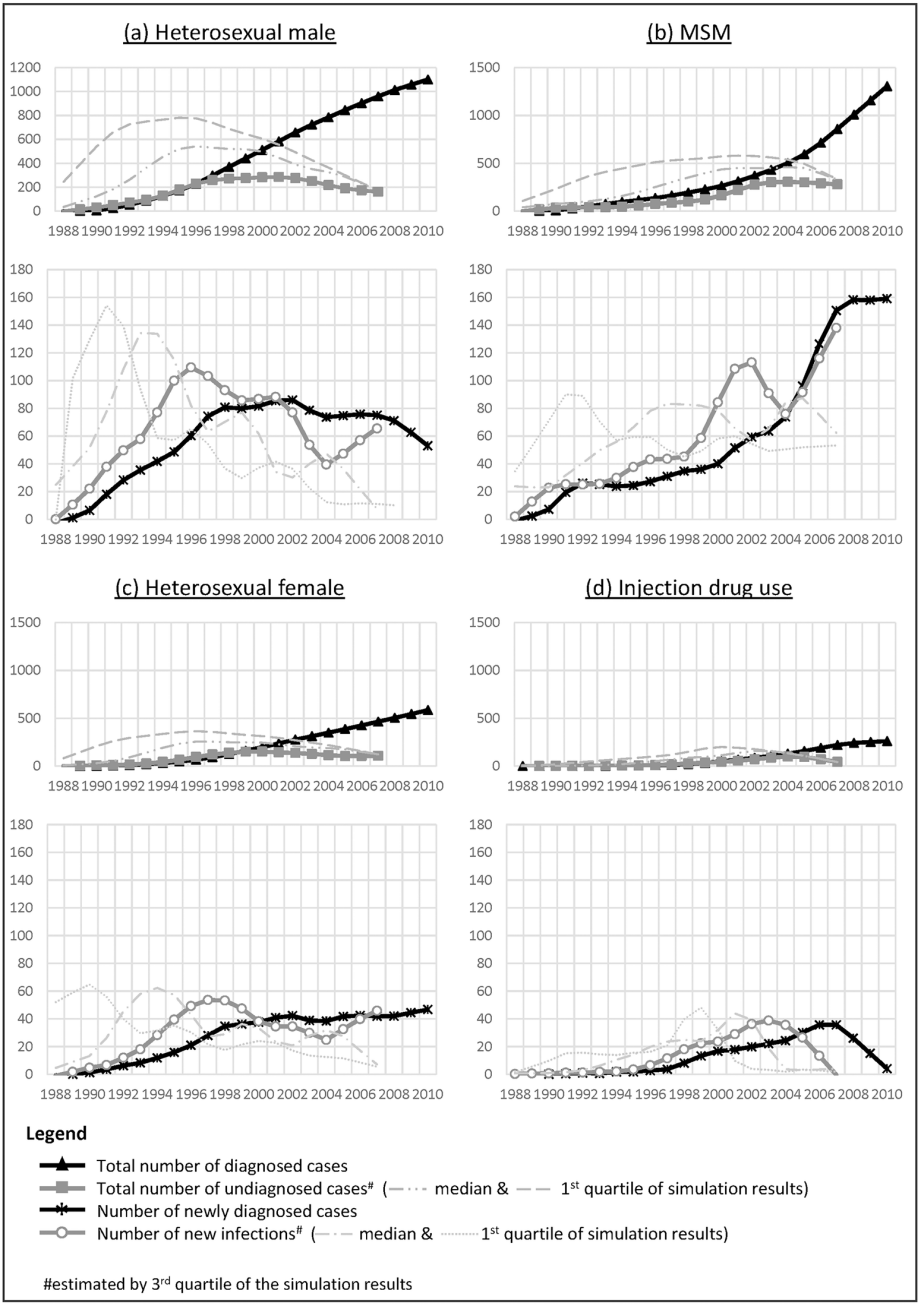
Annual number of reported new diagnoses and prevalent cases (number of diagnosed HIV-infected cases who were alive), estimated new infections and undiagnosed infections (total number of infections remaining undiagnosed) with uncertainty intervals smoothed by Seasonal-Trend Decomposition Procedure based on Loess for (a) heterosexual male, (b) MSM, (c) heterosexual female and (d) IDU.

Compared with the annual new diagnoses, the peak of new infections occurred 4–6 years in advance (Fig 1). The peak for new heterosexual male and female infections was reached in 1996–1998, followed by a decline in 1998–2004. The rebound of new heterosexual male infections after 2004 on the newly constructed curve contrasted significantly with the plateauing of the reported incidence curve from 1998 onward. The annual total number of undiagnosed heterosexual male and female was at least two-folds that of new diagnoses in 1995–2007. The estimated proportion of undiagnosed individuals dropped linearly from 52% in 1996 to 16% in 2006 among heterosexual male, and from 60% to 20% in among heterosexual female.

For men-who-have-sex-with-men (MSM), the epidemic curves were distinctly different between new infections and new diagnoses. (Fig 1) Following a steady increase in the 1980s and 1990s, rapid upsurge of new infections could be seen in 1998–2001, six years in advance of the new diagnoses curve. After a drop in 2001–2004, the MSM epidemic rose again, as shown by the increasing number of new infections. The annual number of undiagnosed MSM was at least twice that of the new diagnoses in 1995–2007, with a widening difference after 1999. The estimated proportion of undiagnosed MSM dropped and became stable at 32%-43% in 1996–2006. The contours of the injection drug users’ (IDU) curves for new infections, new diagnoses and undiagnosed were similar, with a rise followed by a decline. There was however an earlier peak for the former (2003 vs 2007). Similar to the heterosexuals, the proportion of undiagnosed IDU fell from 56% in 1996 to 27% in 2006.

**Fig 2:**
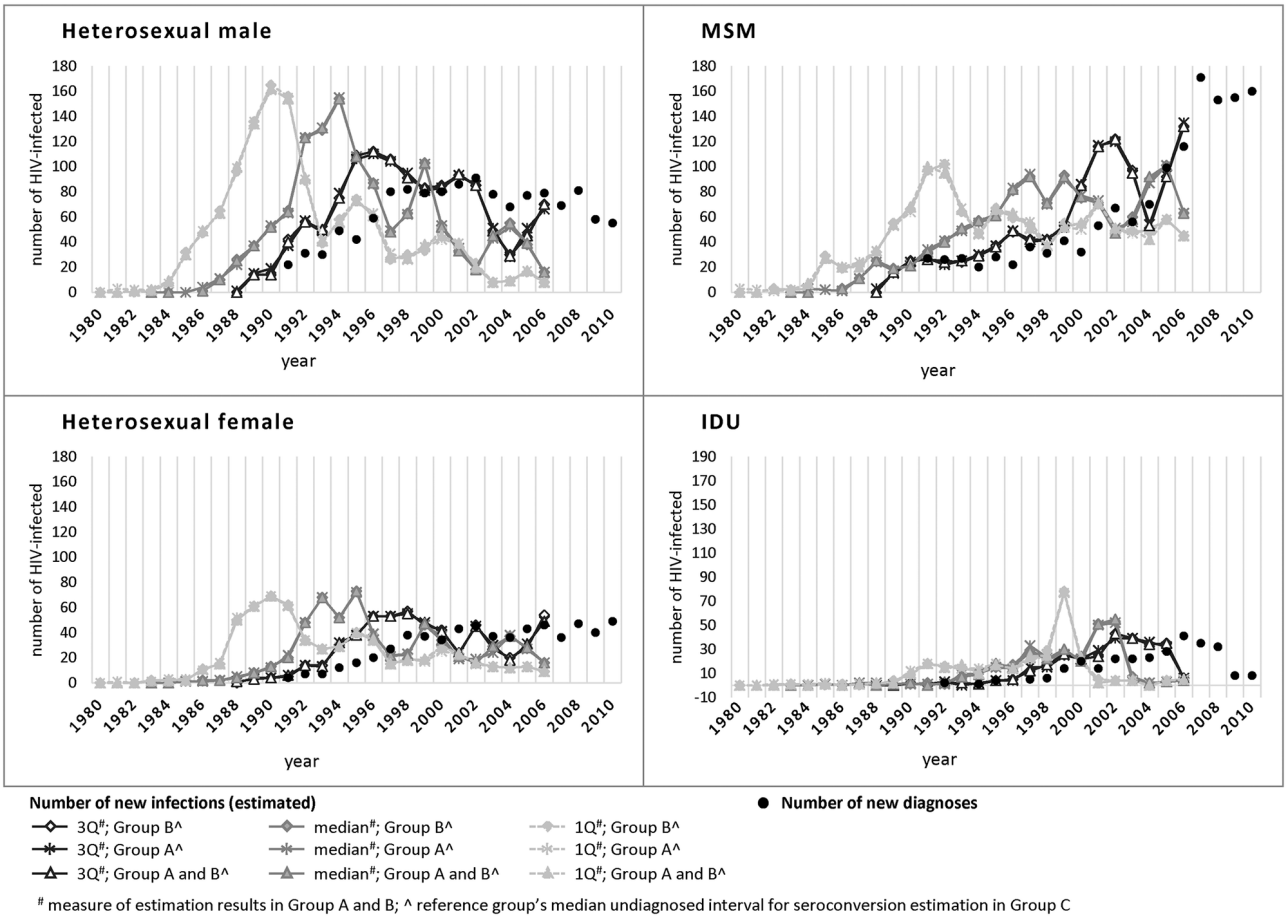
Sensitivity analysis of new infection curves constructed by computing the first quartile, third quartile and median of the simulation results in Group A and B, and the median of undiagnosed interval in reference group (Group A, Group B, Group A and B) for Group C, by mode of transmission.

In the sensitivity analyses, the difference between annual number of new infections using different reference groups (Group A, Group B, or Group A and B) for estimating seroconversion year in Group C were minimal (Fig 2). The contours of new infection curves using different measures (first quartile, median and third quartile) from the 3 groups of patient data were largely similar, though the specific time of rise and fall varied.

### Undiagnosed interval

**Fig 3:**
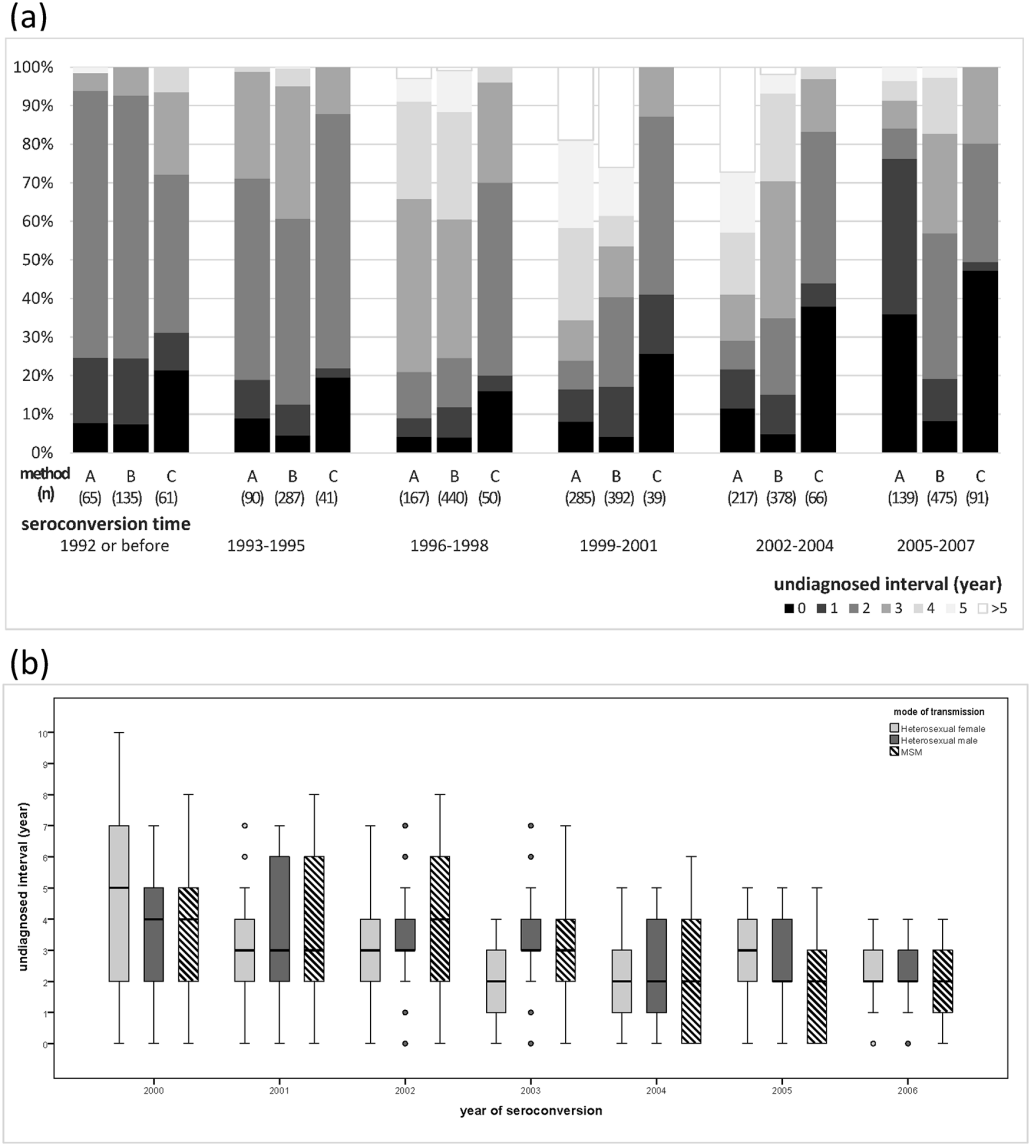
A) Temporal variation of the proportion of undiagnosed intervals (years) and the seroconversion estimation methods applied. B) Yearly variation of undiagnosed intervals (years) by mode of transmission.

The interquartile range of estimated undiagnosed intervals was 1–4 years (range: 0–10 years). The proportion of short undiagnosed intervals (≤2 years) increased across time. Also, the distribution of estimated undiagnosed intervals varied both by the estimation methods and mode of transmission. Higher variation of undiagnosed interval was observed in Group A than the other two groups. (Fig 3a) The proportion of longer undiagnosed interval was higher in Group B than those in Group A, while the proportion in Group C was the lowest. By mode of transmission, the first quartile of undiagnosed interval in MSM was much shorter than that of heterosexual male and female. (Fig 3b) The interquartile range of undiagnosed intervals was at least 3 years in MSM after 1999, while the high variation of intervals among heterosexual male and female patients was seen in 2000–2001 only.

Dichotomizing the undiagnosed intervals by a threshold of 2 years, two groups (>2 years vs ≤2 years) were compared (Table 3). Patients with longer undiagnosed interval were more likely to have contracted HIV through heterosexual contact (OR = 1.61, 95%C.I. = 1.42–1.84), be female (OR = 1.20, 95%C.I. = 1.02–1.43), of Chinese ethnicity (OR = 1.29, 95%C.I. = 1.12–1.49), and age >64 at diagnosis (OR = 2.96, 95%C.I. = 2.05–4.26, aged ≤35 as reference group). At diagnosis, they were more likely to have higher baseline viral load (>log_10_ 5 copies/mL) (aOR = 1.25, 95%C.I. = 1.07–1.47) and to be in late HIV diagnosis (aOR = 2.87, 95%C.I. = 2.45–3.37), after adjusting for age at diagnosis, being MSM and Chinese. Adjusted by the same confounders, clinically, they were more likely to have initiated HAART (aOR = 1.82, 95%C.I. = 1.54–2.14), and be diagnosed with AIDS (aOR = 1.95, 95%C.I. = 1.69–2.25). In the sensitivity analysis, factors associated with undiagnosed interval >2 years as shown in S2 Table were similar to results in Table 3, but gender was no longer significantly associated.

**Table 3:** Characteristics of HIV-infected patients with long (>2 years) undiagnosed interval from estimated seroconversion to HIV diagnosis, compared to patients with a shorter interval.

	Undiagnosed interval ≤2 years (n = 1929)		Undiagnosed interval >2 years (n = 1766)		Crude Odds Ratio (OR)		Adjusted Odds Ratio (aOR)‡	
	no.	%	no.	%	OR	95% CI	aOR	95% CI
**Demographics**								
Gender								
Male	1612	53%	1428	47%		*ref*		
Female	317	48%	338	52%	1.20	1.02–1.43*		
Ethnicity								
Non-Chinese (Asian, White, others, NA)	593	57%	452	43%		*ref*		
Chinese	1336	50%	1314	50%	1.29	1.12–1.49*		
Mode of transmission								
Non-heterosexuals (MSM, IDU, BL and UN)	1036	58%	739	42%		*ref*		
Heterosexuals	893	47%	1027	53%	1.61	1.42–1.84*		
*Heterosexual female*	298	48%	326	52%		*ref*		
*Heterosexual male*	595	46%	701	54%	1.08	0.89–1.3		
Non-MSM (heterosexuals, IDU, BL and UN)	1082	47%	1223	53%		*ref*		
MSM	847	61%	543	39%	0.57	0.5–0.65*		
**Conditions at diagnosis**								
Age at diagnosis								
Aged ≤35	1075	61%	696	39%		*ref*		
Aged 36–64	807	45%	980	55%	1.88	1.64–2.14*		
Aged >64	47	34%	90	66%	2.96	2.05–4.26*		
Baseline VL (copies/mL)								
< = log_10_ 5	823	47%	924	53%		*ref*		*ref*
>log_10_ 5	432	40%	660	60%	1.36	1.17–1.59*	1.25	1.07–1.47*
Late HIV diagnosis^#^								
No	1622	60%	1074	40%		*ref*		*ref*
Yes	307	31%	692	69%	3.4	2.92–3.97*	2.87	2.45–3.37*
**Treatment**								
Initiated HAART by 2012								
No	552	64%	313	36%		*ref*		*ref*
Yes	1377	49%	1453	51%	1.86	1.59–2.18*	1.82	1.54–2.14*
Time from HAART to SVL								
>3 months	783	48%	845	52%		*ref*		*ref*
≤3 months	606	50%	618	50%	0.94	0.81–1.1	0.97	0.83–1.13
**Outcomes**								
Years with NSVL after diagnosis								
< = 3 years	1030	47%	1173	53%		ref		*ref*
>3 years	899	60%	593	40%	0.58	0.51–0.66*	0.66	0.57–0.76*
Ever diagnosed with AIDS								
No	1387	60%	932	40%		*ref*		*ref*
Yes	542	39%	834	61%	2.29	2–2.62*	1.95	1.69–2.25*
Deceased by 2012								
No	1725	54%	1498	46%		*ref*		*ref*
Yes	204	43%	268	57%	1.51	1.25–1.84*	1.03	0.84–1.27

*p-value <0.05

MSM-men who have sex with men, IDU-injection drug use, BL-blood transfusion, UN-undetermined, VL-viral load, HAART- highly active antiretroviral therapy, SVL-suppressed viral load (≤500copies/mL), NSVL-non-suppressed viral load (>500copies/mL)

^‡^adjusted by mode of transmission (MSM vs non-MSM), age at diagnosis (continuous) and ethnicity (Chinese vs non-Chinese) in multivariable logistic regression model

^#^Late HIV diagnosis refers to patients diagnosed with AIDS within 3 months of HIV diagnosis

## Discussion

In this study, we estimated the seroconversion time of individual HIV-infected adults to reconstruct the epidemic curves for Hong Kong, an approach that managed to remove the influence of the highly varied patterns of HIV diagnoses. Compared to a previous local study which used a modified back-calculation method at population level for estimating HIV incidence,[6] our incidence curve showed a similar epidemiologic trend with their estimated curve, but our curve ran 4–5 years ahead of theirs. Apart from the different sources and characteristics of data for estimation, the variation of parameters used could be another reason of discrepancy. In our study, we used pre-treatment CD4 depletion rates to back-calculate the year of seroconversion, while the previous local study was parametrized by the lengths of incubation period, settings of diagnosis (routine testing and symptom-related testing) and the annual number of HIV diagnoses and AIDS diagnoses.[6] Elsewhere, back-calculation of the seroconversion year with CD4 depletion rate has been applied,[12, 13, 15] though most had used the depletion rate of seroconverters as reference group. The latter was adopted as one of the three approaches for estimation in this study. Another approach was applied for patients with very comprehensive CD4 reference data, while a third approach was adopted for patients without any pre-treatment CD4 count. Our mixed-methods approach for estimation illustrated the complexity of data availability in reality, reflecting that a single method might not be able to solve all problems. Our estimated pre-treatment CD4 depletion rate was 56/μL—60/μL per year for sexually acquired HIV infections, which was consistent with the estimated range for patients from Europe and Australia.[27]

Apart from showing an earlier peak in the incidence curves compared to new diagnoses curves, it is noted that the contour of the epidemic curves varied remarkably by the route of HIV transmission. Instead of a continuous rise in the number of newly diagnosed MSM through 2010, our seroconversion curve peaked in 2000–2001, followed by a temporal decline in 2001–2004, and then rose back to the 2001 level. With the increasing number of undiagnosed MSM in the community, the number of newly diagnosed MSM was expected to rise significantly and continuously. However, the observed increase was relatively modest. This could be partly explained by the relatively short undiagnosed interval in HIV-infected MSM. The transmission risk tended to fall after diagnosis as a result of behavioural changes [2] and viral load suppression following HAART.[4] The trend was also consistent with the relatively stable HIV prevalence of MSM reported at different time-points in Hong Kong in 2006, 2008 and 2011.[28]

The epidemic growth for heterosexually acquired infection was very different. The total number of undiagnosed HIV infected heterosexuals increased in 1996–2002, followed by a slower growth afterwards. Unlike MSM, HIV-infected heterosexuals were more likely to have longer undiagnosed interval. This might be partly due to the high proportion of non-locally acquired heterosexual infections,[29] and could also be a result of their different partnership pattern. A Taiwan study on Chinese heterosexual partnership showed that many (around 56%) were in serial monogamy [30], implying on-going transmission could be limited. When a heterosexual person is infected with HIV, virus transmission might occur to affect 1 or 2 sex partners regardless of the length of the undiagnosed interval. This pattern differs considerably from the dense networking of some MSM, whose HIV infection may be characterised by rapid growth.[31] While female sex workers (FSW) could form a bridge in intensifying virus spread, their low HIV prevalence of below 0.2%,[32, 33] means that rapid dissemination from HIV infected FSW to their clients was unlikely.

For IDU, the flat contour of newly infected IDU lent support to the uncommon occurrence of local transmission, which could be attributed to the longstanding methadone treatment programme introduced since the 1970s.[34] For both heterosexuals and IDU, the epidemic curves redrawn with estimated seroconversion years ran 4 years in advance of their diagnoses. Evidently, the estimation of new infections has contributed to the assessment of HIV transmission risk in the population and subpopulations.

The length of undiagnosed intervals carries significant public health implications. Our study has identified the following associating factors with long undiagnosed interval in Hong Kong: heterosexual, female, Chinese ethnicity, and age >64 at diagnosis. The longer undiagnosed interval in heterosexual male/female and elderly was probably related to their lower perceived risks of infection, compared to younger MSM in the local community, an observation that has also been made elsewhere.[35] Because of the low perceived risk of infection, HIV testing experiences of heterosexuals, especially the elderly, are predictably low. With 27% of patients in late HIV diagnosis, expanded HIV testing is therefore one most imminent strategy for shortening the undiagnosed interval, which can serve not just to improve clinical outcomes but also reduce transmission risk in the community. An earlier diagnosis of HIV infection is desirable regardless of the route of transmission. Our results showed that early diagnosis was negatively associated with high baseline viral load and AIDS diagnosis, adjusted by ethnicity, mode of transmission and age at diagnosis. Paradoxically, they had a longer duration of non-suppressed viral load, which was probably due to higher baseline CD4 and therefore longer interval between diagnosis and treatment initiation, in accordance with the CD4-guided approach to HAART. Expanded treatment initiation has recently been recommended by World Health Organization to cover all diagnosed patients with any CD4 count,[36] a strategy supported by increasing number of clinical studies which would predictably shorten the duration of non-suppressed viral load if early diagnosis could be achieved. Separately, a meta-analysis study concluded that there was a lower prevalence of risk behaviour after one became aware of the HIV status.[2] A combination of early diagnosis and universal treatment without regard to the prevailing CD4 count would predictably reduce the undiagnosed interval and facilitate the plateauing of the HIV epidemic curves.

We acknowledge that the study carries some limitations. As it takes time to capture a majority of the infected patients in a year, estimation by back-calculation approach was limited to the use of data before 2008, five years before the end of our data collection period. The estimation for patients in Group C was based on the estimation result in Group B, which might not be the best reference group to represent patients in Group C. However, from the sensitively analyses for reference group selections, Group B might be a better option under existing data availability. Also, in the course of conducting CD4 depletion rate estimation, the small proportion of rapid progressors and long-term non-progressors had been ignored. Their contribution to the epidemic growth is however predicted to be small. We assumed linear trajectory of pre-treatment CD4 decline and ignored the association between CD4 and viral load levels. We acknowledge there exists a wide range of uncertainties in applying our proposed estimation methods, and there are rooms for improvement in future epidemiologic estimation. Finally, it may not be easy to have Group A method regularly applied as the immediate treatment strategy is becoming a routine in clinical services. In practice, the future approach might rely more on Group B and C with additional information such as patients’ perception of their infection date, viral load measurement, seroconversion illnesses and molecular study. Nonetheless, we believe our mixed-methods approach has demonstrated the feasibility of using readily available longitudinal clinical data for epidemiologic estimation.

In conclusion, we have endeavoured to estimate the undiagnosed interval at individual level with currently available clinical data, without resorting to sophisticated laboratory technologies, to reconstruct HIV epidemic curves founded on seroconversion time rather than clinical diagnoses. The study was conducted in a small city where a majority of patients were managed according to similar clinical guidelines. In principle, a plausible estimation approach can be adopted, which can be turned into a public health tool for describing and projecting HIV epidemiology in places where pre-treatment CD4 data are available from the HIV services.

## Supporting Information

S1 Table The central tendency of discrepancy between simulation results in Group A and mid-point interval of seroconverters. (PDF)

S1 Fig Distribution of 3rd quartile of simulation results for seroconversion year estimation in Group A and Group B. (TIF)

S2 Table Characteristics of HIV-infected patients with long (>2 years) undiagnosed interval from estimated seroconversion to HIV diagnosis in sensitivity analysis, compared to patients with a shorter interval. (PDF)
