# Supplementary material for: Estimation of the Undiagnosed Intervals of HIV-Infected Individuals by a Modified Back-Calculation Method for Reconstructing the Epidemic Curves
Source: PLoS One. 2016 Jul 12;11(7):e0159021. doi: 10.1371/journal.pone.0159021 (PMC4942036; doi:10.1371/journal.pone.0159021)
Supplement: S1 Table — (PDF) [file pone.0159021.s001.pdf]

S1 Table. The central tendency of discrepancy between simulation results in Group A and mid-point interval of seroconverters

	Central tendency of discrepancy (years)	
**Discrepancy between mid-point and:**	**Median**	**IQR**
**Overall**		
estimated mean of simulation results	7	6-8
estimated median of simulation results	6	5-7
estimated 3rd quartile of simulation results	3	2-4
estimated 1st quartile of simulation results	10	9-12
**CD4 at diagnosis >500/µL**		
estimated mean of simulation results	7	5-8
estimated median of simulation results	6	4-7
estimated 3rd quartile of simulation results	3	2-4
estimated 1st quartile of simulation results	10	8-12
**CD4 at diagnosis <=500/µL**		
estimated mean of simulation results	7	6-8
estimated median of simulation results	6	4-7
estimated 3rd quartile of simulation results	3	2-4
estimated 1st quartile of simulation results	11	9-12
