# Supplementary figures and images for: Estimation of the Undiagnosed Intervals of HIV-Infected Individuals by a Modified Back-Calculation Method for Reconstructing the Epidemic Curves
Source: PLoS One. 2016 Jul 12;11(7):e0159021. doi: 10.1371/journal.pone.0159021 (PMC4942036; doi:10.1371/journal.pone.0159021)

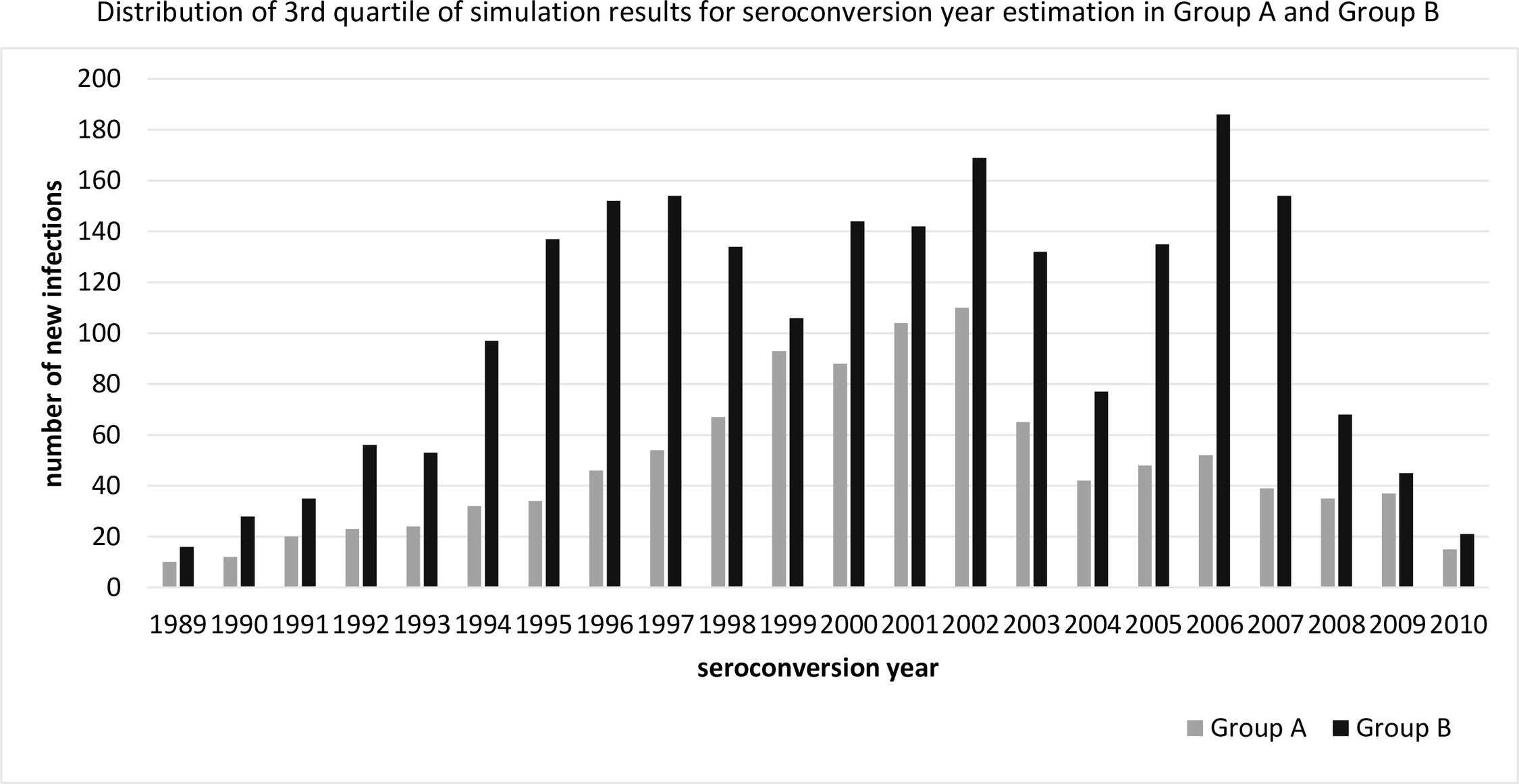

Supplement: S1 Fig — (TIF) [file pone.0159021.s002.tif]
