# Supplementary material for: Estimation of the Undiagnosed Intervals of HIV-Infected Individuals by a Modified Back-Calculation Method for Reconstructing the Epidemic Curves
Source: PLoS One. 2016 Jul 12;11(7):e0159021. doi: 10.1371/journal.pone.0159021 (PMC4942036; doi:10.1371/journal.pone.0159021)
Supplement: S2 Table — (PDF) [file pone.0159021.s003.pdf]

S2 Table Characteristics of HIV-infected patients with long (>2 years) undiagnosed interval from estimated seroconversion to HIV diagnosis in sensitivity analysis, compared to patients with a shorter interval

	**undiagnosed period ≤2 years (n=1666)**		**undiagnosed period >2 years (n=2029)**		**Crude Odds Ratio**	
	**n**	**%**	**n**	**%**	**OR**	**95% CI**
**Demographics**						
Gender						
Male	1385	46%	1655	54%		
Female	281	43%	374	57%	1.11	0.94-1.32^
Ethnicity						
Non-Chinese (Asian, White, others, NA)	517	49%	528	51%		
Chinese	1149	43%	1501	57%	1.28	1.11-1.48*^
Mode of transmission						
Non-heterosexuals (MSM, IDU, BL and UN)	922	52%	853	48%		
Heterosexuals	744	39%	1176	61%	1.71	1.5-1.95*^
Heterosexual female	262	42%	362	58%		
Heterosexual male	482	37%	814	63%	1.22	1.01-1.49*
Non-MSM (heterosexuals, IDU, BL and UN)	866	38%	1439	62%		
MSM	800	58%	590	42%	0.44	0.39-0.51*^
**Conditions at diagnosis**						
Age at diagnosis						
aged ≤35	977	55%	794	45%		
aged 36-64	658	37%	1129	63%	2.11	1.85-2.41*^
aged >64	31	23%	106	77%	4.21	2.79-6.35*^
Baseline VL (copies/mL)						
<=$\log_{10}$ 5	742	42%	1005	58%		
>$\log_{10}$ 5	305	28%	787	72%	1.91	1.62-2.24*^
Late HIV diagnosis[#]						
no	1597	59%	1099	41%		
yes	69	7%	930	93%	19.59	15.16-25.31*^
**Treatment**						
Initiated HAART by 2012						
no	501	58%	364	42%		
yes	1165	41%	1665	59%	1.97	1.69-2.3*^
Time from HAART to SVL						
>3 months	652	40%	976	60%		
≤3 months	525	43%	699	57%	0.89	0.77-1.03

**Outcomes**						
Years with NSVL after diagnosis						
<=3 years	852	39%	1351	61%		
>3 years	814	55%	678	45%	0.53	0.46-0.6*^
Ever diagnosed with AIDS						
no	1375	59%	944	41%		
yes	291	21%	1085	79%	5.43	4.66-6.33*^
Deceased by 2012						
no	1519	47%	1704	53%		
yes	147	31%	325	69%	1.97	1.6-2.42*^

*p-value <0.05 in this table

^p-value <0.05 in Table 3

MSM-men who have sex with men, IDU-injection drug use, BL-blood transfusion, UN-undetermined, VL-viral load, HAART- highly active antiretroviral therapy, SVL-suppressed viral load (≤500copies/mL), NSVL-non-suppressed viral load (>500copies/mL)

Late HIV diagnosis refers to patients diagnosed with AIDS within 3 months of HIV diagnosis
